# Can probiotic supplements improve the symptoms of autism spectrum disorder in children? A protocol for systematic review and meta analysis

**DOI:** 10.1097/MD.0000000000018621

**Published:** 2021-03-12

**Authors:** Kai Feng, Ying Zhao, Qingyang Yu, Jialin Deng, Jingjing Wu, Lingjia Liu

**Affiliations:** a Beijing University of Chinese Medicine, Beijing; b Chengdu University of Chinese Medicine, Chengdu; c Department of Pediatrics, Dongzhimen Hospital, Beijing University of Chinese Medicine, Beijing, China.

**Keywords:** adolescent, autism spectrum disorder, children, probiotic supplements

## Abstract

**Introduction:**

Autism spectrum disorder (ASD) is a neurodevelopmental disorder with increasing incidence. The externalizing and internalizing problems among children with ASD often persistent and highly impair functioning of both the child and the family. Children with ASD often develop gut-related comorbidities and dysbiosis can have negative effects on not only the gastrointestinal (GI) tract, but also psychological symptoms. Dietary exclusions and probiotic supplements also have been investigated in the management of ASD symptoms. Especially, there is some anecdotal evidence that probiotics supplements are able to alleviate GI symptoms as well as improve behaviors in children with ASD.

**Method and analysis:**

This review will report on overall studies that include randomized control trials, randomized cross-over studies and cluster-randomized trials designs that consider curative effect in children with ASD by probiotic supplements. We will search 6 databases: MEDLINE, Embase, Scopus, PubMed, The Cochrane Library, and Web of Science and we will perform a manual search the journal Autism and information of ongoing or unpublished studies. The Mixed Methods Appraisal Tool (MMAT) will be used to assess quality of articles and the Jadad scale will be used to assess for bias. Assessment of publication bias will be performed using funnel plots generated by Comprehensive Meta-Analysis (CMA) 3.0 software. Clarifying the evidence in this area will be important for future research directions when reformulating and promoting the therapeutic regime in the field.

**Ethics and dissemination:**

There are no human participants, data, or tissue being directly studied for the purposes of the review; therefore, ethics approval and consent to participate are not applicable. The results of this study will be presented at conferences and published in peer-reviewed journals.

**Registration and status:**

PROSPERO 2019 CRD42019132754.

## 1 Introduction

Autism spectrum disorder (ASD) is a group of complex childhood-onset neurodevelopmental disorder marked by persistent deficits in social communication or interaction skills along with restricted interests and repetitive behaviors.^[1]^ Approximately 1 in 68 children are characterized by ASD according to estimates from Centers for Disease Control and Prevention (CDC)'s Autism and Developmental Disabilities Monitoring (ADDM) Network.^[2]^ Systematic reviews around the world estimated different global prevalence rates of ASD between 14.8 per 10,000 and 7.6 per 1000.^[3–7]^ Further, it is mainly diagnosed during the childhood developmental stage around the age of 4 to 5^[8]^ and more commonly in males than females, with a 4:1 male-to-female ratio.^[7]^ ASD is typically diagnosed using the criteria from the Diagnostic and Statistical Manual of Mental Disorders, 5th edition: DSM-5.^[1]^ Both externalizing problems (such as disruptive behavior, aggression, and self-injury) and internalizing problems (such as depression and anxiety) are more common among children with ASD than among typically developing children. These conditions are often persistent^[9]^ and highly impair functioning of both the child and the family.^[10]^ Despite many years of vast study, the causes of ASD are still unknown. Various risk factors including genetic,^[11]^ infectious,^[12]^ metabolic,^[13]^ immunological,^[14]^ environmental,^[15]^ family relationship,^[16]^ and nutritional^[17]^ have been investigated; however, the relationship between gut microbiota and ASD has not received sufficient attention.

People with ASD develop several comorbidities, including gut-related comorbidities such as diarrhea, constipation, commutative diarrhea/constipation, abdominal pain, vomiting, reflux, or bloating are quite common, and they are correlated with the severity of the neurobehavioral disorder.^[18]^ In this sense, people with ASD with gastrointestinal (GI) symptoms exhibit more anxiety problems and other somatic complaints, together with less social interaction than ASD people without GI symptoms.^[19]^ In addition, the severity of GI symptoms who with ASD have been linked with derangements in the gut microbiota, such as during administration of antibiotics. Curiously, the GI and behavioral symptoms reverted once antibiotics were stopped.^[20]^ This opens up further avenues of research for the role of gut microbiota-altering agents such as probiotics as a potential therapeutic option. Probiotics aim to restore normal balance of human gut microbiota and recent studies have suggested that probiotics are beneficial in remedying several psychological symptoms such as anxiety and depression.^[21]^ It is theorized that there exists a complex interplay between the GI tract and the brain termed the “gut–brain axis.” Gut microbiota play an important role in modulating this “gut–brain axis” and dysbiosis can have negative effects on not only the GI tract, but also psychological symptoms.^[22]^ Dietary exclusions and probiotic supplements have been investigated in the management of ASD symptoms.^[23]^ Especially, there is some anecdotal evidence that probiotics supplements are able to alleviate GI symptoms as well as improve behaviors in children with ASD.^[24]^

Despite significant public interest in probiotic supplements, systematic reviews of the effect of probiotic supplements on ASD and its associated behavioral/GI symptoms are lacking. Therefore, the research team viewed this as a critical research area.

## 2 Objectives

The holistic objective is to undertake a systematic review and meta-analysis to answer the questions:

1. Can probiotic supplements improve the core symptoms in children with ASD?
2. Is there any significant difference in the rates of probiotic supplements prescribed for children with ASD worldwide?
3. What is the prevalence of individuals with ASD receiving and not receiving probiotic supplements treatment?

## 3 Method

Reporting will be developed on the basis of the Preferred Reporting Items for Systematic Reviews and Meta-Analyses (PRISMA) statement.^[25]^ This review has been registered on the PROSPERO, ID: CRD42019132754.

### 3.1 Eligibility criteria of studies

#### 3.1.1 Inclusion criteria

1. Study will include a primary diagnosis of ASD.
2. The participants are restricted to be children and adolescents under the age of 18.
3. Study will include randomized control trials, randomized cross-over studies and cluster-randomized trials.
4. There will be no language restrictions and no published data will be included.

#### 3.1.2 Exclusion criteria

1. If participants with other neuropsychiatric diseases.
2. If participants were in a transient state, such as infection phase or pregnancy.
3. The interventions in control group of studies were not placebo.
4. Conference proceedings, editorials, commentaries, and book chapters/book reviews will be excluded.

### 3.2 Outcomes

The primary outcome will be to identify if probiotic supplements can improve the symptoms of autism spectrum disorder in children. From the outcome identified, the probiotic supplements that need further support to better extend to remedy ASD.

### 3.3 Information sources and search strategy

There will be no language restrictions. The study will perform a literature search using 6 databases: MEDLINE, Embase, Scopus, PubMed, The Cochrane Library, and Web of Science. The search strategy was developed to capture all studies that would meet the above eligibility criteria and utilize appropriate journals. The database search strategies were developed with the assistance of a university librarian, who will work along with the authors to develop the search strategy, with experience in systematic reviews. To achieve the intended purpose of the objective, searches will be performed by one of the authors, who completed the systematic review and meta-analysis training of West China Hospital, Sichuan University, to develop the search strategy. The search strategy will include only search terms related to “ASD” and “probiotic supplements” and adapted for each database as necessary. Studies published until the date the searches executed will be sought. The full search strategy is included in Table 1.

**Table 1:** Key terms for database search strategy.

Step	Query
#1	(pervasive developmental disorder^∗^ OR neurodevelopmental disorder^∗^ OR Asperger OR autism spectrum disorder^∗^ OR autism OR autistic OR ASD)
#2	(microbio^∗^ OR Probiotic^∗^ OR Bacteroides OR Desulfovibrio OR Clostridium OR Blautia OR Dialister OR Prevotella OR Veillonella OR Turicibacter OR Bifidobacteri^∗^ OR Lactis OR Lactobacillus OR Lacto^∗^ OR Bacillus^∗^ OR yogurt OR Escherichiacoli OR Saccharomyces)
#3	(randomized controlled trial OR controlled clinical trial OR randomized OR placebo OR drug therapy OR randomly OR trial OR groups OR RCT) NOT (animals)
#4	#1 + #2 + #3

ASD = autism spectrum disorder.

And the journal Autism will be hand searched. Analogously, we will perform a manual search in the reference list of systematic reviews and meta-analysis on ASD treatment by probiotic supplements retrieved in the database search. Moreover, we will contact each included study author to consult basic data and we will search the https://clinicaltrials.gov/ and http://apps.who.int/trialsearch in order to reach information of ongoing or unpublished studies.

### 3.4 Identification and selection of studies

The titles and abstracts of all studies generated through the combined database searches will be merged using Endnote X8 remove the duplicates. Two authors (FK and ZY) will independently screen search results against eligibility criteria. All studies that meet the eligibility criteria on screening titles and abstracts will be sourced and read in full. Two other authors will review and screen the titles and the results and compare with the eligibility criteria, to increase validity (DJL and WJJ).^[26]^ We will resolve any disagreements though discussion (FK, ZY, DJL, WJJ, YQY). The search strategy and study selection processes will be documented using a PRISMA study flow diagram.^[27]^

### 3.5 Data extraction

One author (LLJ) will independently extract data from selected studies on key components addressing our research questions. A standardized data extraction form will be used to extract data from the included studies for assessment of quality and evidence synthesis. The following information will be extracted:

1. Study data (1st author, year of publication, country, a journal of publication, study period).
2. Study design (a type of research, details of randomized control trial, randomized cross-over studies or cluster-randomized trials and the validity of confirmative diagnosis, and method of data collection).
3. Characteristics of study participants (condition, age, gender, race, sample size, and sampling procedures).
4. Probiotic supplements interventions characteristics (formulation, dose range, dose, frequency, duration).
5. Control interventions characteristics (formulation, dose range, dose, frequency, duration).
6. Outcome measures: Questionnaires, such as CGI-S, CGI-I, ADI-R, ABC-T, CBCL, SRS, SNAP-IV, and so on.

### 3.6 Assessing of study quality

Risk of bias of included studies will be assessed using the Cochrane Risk of Bias Assessment tool that contains several items under 7 categories such as random sequence generation, allocation concealment, blinding of participants and investigators, the blindness of outcome assessments, incomplete outcome data, selective outcome reporting, and other biases. Based on the assessment, the studies will be evaluated as low, unclear, or high bias. The Jadad scale will be used to evaluate the quality of each trial where 3 domains in the scale cover randomization (0–2 points), blinding (0–2 points), and dropouts and withdrawals (0–1 point).^[28]^ A trial with a score 2 indicates low quality while a score of ≥3 indicates high quality. Assessment of publication bias will be performed using funnel plots generated by Comprehensive Meta-Analysis (CMA) 3.0 software.

### 3.7 Data synthesis

Search results will be summarized in a PRISMA Protocol. First, the studies will be categorized according to study design and then the study characteristics.

For quantitative studies: Frequencies and percentages will be reported for categorical variables and the means and standard deviations for continuous variables, depending on the data.^[29,30]^ Where appropriate, pooling of data and meta-analysis will be performed.^[31,32]^For qualitative studies: we anticipate the qualitative data will describe the perspectives of patients with ASD and the barriers and enablers they may identify. The qualitative data will be analyzed using the Mixed Methods Appraisal Tool (MMAT),^[33,34]^ identifying patterns (themes) within the data. Researchers will determine categories based on the analysis.

Where there is unreported data and/or clarification is required to determine if the study can be included, we (YQY) will attempt to contact the study authors, to obtain the missing data using a maximum of 3 emails. If data cannot be obtained, we will analyze the available data and in the discussion section, report the potential impact.

Publication bias will be explored through the visual inspection of the funnel plot asymmetry, and Egger's linear regression test.^[35]^

## 4 Ethics and dissemination

There are no human participants, data, or tissue being directly studied for the purposes of the review; therefore, ethics approval and consent to participate is not applicable. None declare conflict of interest. The results of this study will be presented at conferences and published in peer-reviewed journals.

## Author contributions

LLJ conceived the study and drafted the protocol. FK, ZY, YQY, DJL, and WJJ contributed to the protocol design.

LLJ, FK, and ZY conceived the search strategy. All authors contributed to the development of inclusion and exclusion criteria. All authors read, contributed, and approved the final manuscript.

**Conceptualization:** Liu Lingjia, Feng Kai, Zhao Ying.

**Investigation:** Feng Kai, Zhao Ying, Yu Qingyang, Deng Jialin, Wu Jingjing.

**Methodology:** Liu Lingjia, Feng Kai, Zhao Ying.

**Project administration:** Liu Lingjia.

**Writing – original draft:** Liu Lingjia, Feng Kai, Zhao Ying.

**Writing – review & editing:** Yu Qingyang, Deng Jialin, Wu Jingjing.
